# Establishing connectivity through microdissections of midbrain stimulation-related neural circuits

**DOI:** 10.1093/brain/awae173

**Published:** 2024-05-29

**Authors:** Georgios P Skandalakis, Clemens Neudorfer, Caitlin A Payne, Evalina Bond, Armin D Tavakkoli, Jessica Barrios-Martinez, Anne C Trutti, Christos Koutsarnakis, Volker A Coenen, Spyridon Komaitis, Constantinos G Hadjipanayis, George Stranjalis, Fang-Cheng Yeh, Layla Banihashemi, Jennifer Hong, Andres M Lozano, Michael Kogan, Andreas Horn, Linton T Evans, Aristotelis Kalyvas

**Affiliations:** Section of Neurosurgery, Dartmouth Hitchcock Medical Center, Lebanon, NH 03756, USA; Department of Neurosurgery, National and Kapodistrian University of Athens Medical School, Evangelismos General Hospital, Athens 10676, Greece; Center for Brain Circuit Therapeutics Department of Neurology Brigham & Women’s Hospital, Harvard Medical School, Boston, MA 02115, USA; MGH Neurosurgery & Center for Neurotechnology and Neurorecovery (CNTR) at MGH Neurology Massachusetts General Hospital, Harvard Medical School, Boston, MA 02114, USA; Movement Disorder and Neuromodulation Unit, Department of Neurology, Department of Neurology, Charité—Universitätsmedizin Berlin, Corporate member of Freie Universität Berlin and Humboldt-Universität zu Berlin, 10117 Berlin, Germany; Section of Neurosurgery, Dartmouth Hitchcock Medical Center, Lebanon, NH 03756, USA; Section of Neurosurgery, Dartmouth Hitchcock Medical Center, Lebanon, NH 03756, USA; Section of Neurosurgery, Dartmouth Hitchcock Medical Center, Lebanon, NH 03756, USA; Department of Neurological Surgery, University of Pittsburgh, Pittsburgh, PA 15213, USA; Integrative Model-Based Cognitive Neuroscience Research Unit, University of Amsterdam, Amsterdam 15926, The Netherlands; Department of Neurosurgery, National and Kapodistrian University of Athens Medical School, Evangelismos General Hospital, Athens 10676, Greece; Department of Stereotactic and Functional Neurosurgery, Medical Center of the University of Freiburg, Freiburg 79106, Germany; Medical Faculty of the University of Freiburg, Freiburg 79110, Germany; Center for Deep Brain Stimulation, Medical Center of the University of Freiburg, Freiburg 79106, Germany; Queens Medical Center, Nottingham University Hospitals NHS Foundation Trust, Nottingham NG7 2UH, UK; Department of Neurological Surgery, University of Pittsburgh, Pittsburgh, PA 15213, USA; Department of Neurosurgery, National and Kapodistrian University of Athens Medical School, Evangelismos General Hospital, Athens 10676, Greece; Department of Neurological Surgery, University of Pittsburgh, Pittsburgh, PA 15213, USA; Department of Psychiatry, University of Pittsburgh, Pittsburgh, PA 15213, USA; Department of Bioengineering, University of Pittsburgh, Pittsburgh, PA 15213, USA; Section of Neurosurgery, Dartmouth Hitchcock Medical Center, Lebanon, NH 03756, USA; Division of Neurosurgery, University Health Network, University of Toronto, Toronto, ON M5T 1P5, Canada; Department of Neurosurgery, University of New Mexico School of Medicine, Albuquerque, NM 87106, USA; Center for Brain Circuit Therapeutics Department of Neurology Brigham & Women’s Hospital, Harvard Medical School, Boston, MA 02115, USA; MGH Neurosurgery & Center for Neurotechnology and Neurorecovery (CNTR) at MGH Neurology Massachusetts General Hospital, Harvard Medical School, Boston, MA 02114, USA; Movement Disorder and Neuromodulation Unit, Department of Neurology, Department of Neurology, Charité—Universitätsmedizin Berlin, Corporate member of Freie Universität Berlin and Humboldt-Universität zu Berlin, 10117 Berlin, Germany; Section of Neurosurgery, Dartmouth Hitchcock Medical Center, Lebanon, NH 03756, USA; Division of Neurosurgery, University Health Network, University of Toronto, Toronto, ON M5T 1P5, Canada

**Keywords:** deep brain stimulation, ventral tegmental area, fibre tractography, neuropsychiatric neural circuits

## Abstract

Comprehensive understanding of the neural circuits involving the ventral tegmental area is essential for elucidating the anatomofunctional mechanisms governing human behaviour, in addition to the therapeutic and adverse effects of deep brain stimulation for neuropsychiatric diseases. Although the ventral tegmental area has been targeted successfully with deep brain stimulation for different neuropsychiatric diseases, the axonal connectivity of the region is not fully understood.

Here, using fibre microdissections in human cadaveric hemispheres, population-based high-definition fibre tractography and previously reported deep brain stimulation hotspots, we find that the ventral tegmental area participates in an intricate network involving the serotonergic pontine nuclei, basal ganglia, limbic system, basal forebrain and prefrontal cortex, which is implicated in the treatment of obsessive–compulsive disorder, major depressive disorder, Alzheimer’s disease, cluster headaches and aggressive behaviours.

## Teaser

Using fibre microdissections in postmortem human brains and population-based fibre tractography, Skandalakis *et al.* show that the ventral tegmental area participates in an intricate network involving limbic, striatal, basal forebrain, and prefrontal circuits. These findings can inform personalized DBS approaches for neuropsychiatric disorders.

## Introduction

The ventral tegmental area (VTA) is a midbrain region containing a diverse population of dopamine-, glutamate- and GABA-releasing neurons.^1^ Apart from synaptic neurotransmitter release, VTA neurons have demonstrated the ability to release neurotransmitters from their cell bodies and dendrites to modulate dopamine-dependent behaviours.^2^ The VTA supports limbic, motor and high-order functions, and its activity is orchestrated by reward- and social-related stimuli.^3-7^ Over the past two decades, the VTA has been used successfully as a deep brain stimulation (DBS) target for neuropsychiatric diseases.^8^ Nonetheless, neither the connections of the human VTA nor the underlying circuits enabling the neuromodulatory effects of DBS of the region are fully understood.^9,10^

The prevalent substrate suggested to facilitate therapeutic outcomes of VTA region DBS is the superolateral medial forebrain bundle (slMFB).^9,11-13^ The slMFB has been reported as a segment of the medial forebrain bundle (MFB) interconnecting the VTA, nucleus accumbens (NAc) and prefrontal cortex (PFC).^14^ Human data regarding the slMFB originate from diffusion MRI (dMRI) studies and are inconsistent in terms of its connectivity.^10^ Animal studies report more extensive connectivity of the MFB with regions such as globus pallidus (GP), amygdala, hippocampal region and entorhinal cortex.^15^ Cross-species MFB discrepancies, in the absence of human histological evidence, have led to criticism of the slMFB^10^ and have raised questions regarding the underlying pathways facilitating VTA DBS.^9^

Detailed anatomical knowledge is crucial for selection of DBS targets related to improved outcomes and precise lead placement.^16,17^ Here, we used high-resolution dMRI datasets and averaged templates from multiple databases of a total *n* ≈ 1100 healthy subjects and cadaveric brains [Duke, Massachusetts General Hospital (MGH), Human Connectome Project (HCP) developmental, HCP healthy adult, HCP ageing] to elucidate further the organization of circuits related to DBS of the VTA region through high-definition fibre tractography guided by cadaver fibre microdissections. We hypothesized that the tracts we characterized are implicated in midbrain and diencephalic DBS. Hence, we used Lead-DBS to analyse previously reported DBS parameters and identify DBS targets implicating the tracts we characterized.

## Materials and methods

**Figure 1:**
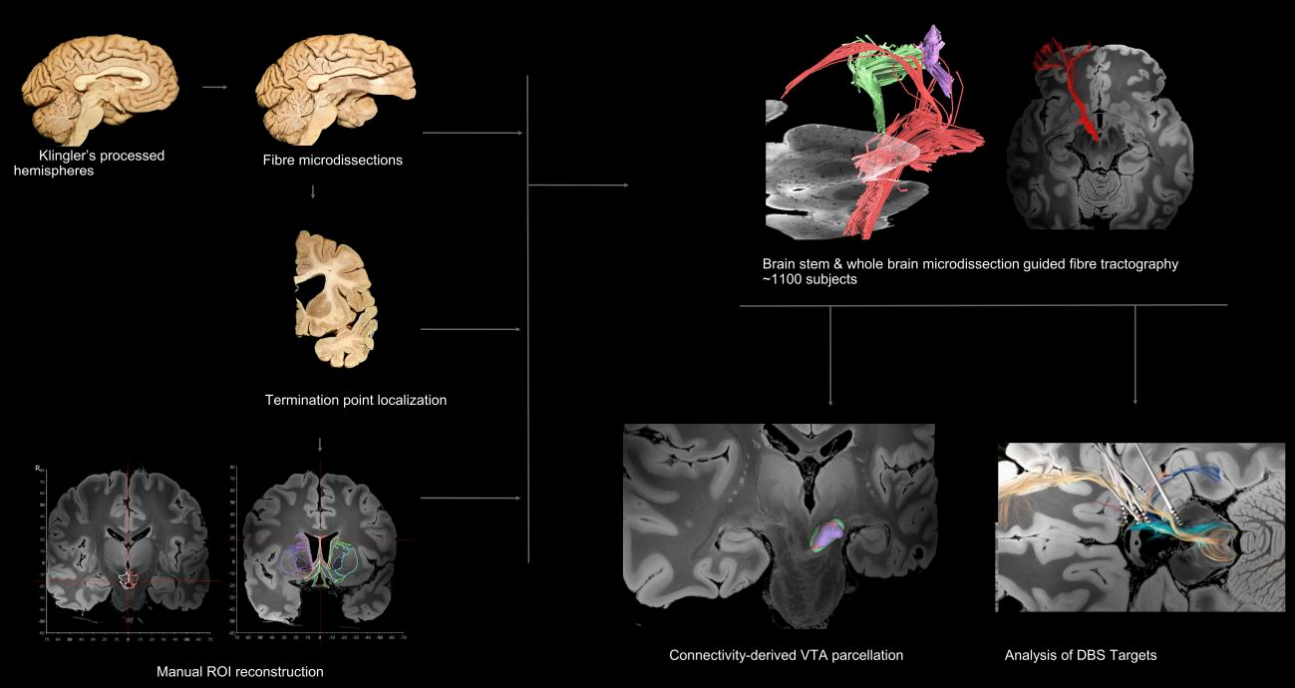
**Illustration of the methods followed in this study.** Initial characterization of ventral tegmental area (VTA)-related fibre tracts and their termination points was achieved through stepwise microdissections in cadaveric hemispheres. Regions of intetest (ROIs) were reconstructed manually for the VTA and recorded termination points. Reconstruction of the VTA tract was achieved through microdissection-guided fibre tractography in multiple databases. A connectivity-driven parcellation of the VTA was performed in an averaged template generated from data of 1065 healthy adults. Lead-DBS was used to study the relationship between the tracts we characterized anatomically and the previously used deep brain stimulation (DBS) targets.

This study comprised multiple layers (Fig. 1). Initial characterization of VTA-related fibre tracts was performed through stepwise microdissections of cadaveric hemispheres. Fibre tractography through a single VTA region of interest (ROI) approach was performed in multiple databases guided by the results of our microdissections. Multiple fibre-tracking studies were performed per subject/averaged template. We used a two-ROI approach for confirmation of our fibre-tractography results: the first ROI was placed on the VTA and the second was used for all identified termination points. A connectivity-driven parcellation of the VTA was performed on an averaged template generated from data of 1065 healthy adults. Lead-DBS was used to study the relationship between the tracts we characterized anatomically and the previously used DBS targets. The fibre-microdissection study has received approval from the Bioethics Committee of University of Athens (protocol number: 118/21.05.2019).

### White matter dissections

Ten normal adult cadaveric formalin-fixed hemispheres were treated according to the Klingler’s preparation and subsequently studied using the white matter microdissection technique. Fibre microdissections were performed with the use of surgical microscopes (OPMI Carl Zeiss, Leica M320) and micro-neurosurgical tools as previously described.^18,19^ The VTA was delineated as the region bounded by the midline medially, substantia nigra anteriorly, red nucleus posteriorly and laterally, subthalamic nucleus superiorly and laterally, and pons inferiorly.^20^

### Tractography

#### Regions of interest

We used the MGH single subject 100 micron MRI dataset^21^ and Duke single brainstem/diencephalon 50/200 μm MRI dataset^22^ to delineate the ROIs for the VTA, raphe nuclei, hypothalamus, mammillary bodies, septal nuclei, bed nucleus of stria terminalis (BNST), nucleus basalis of Meynert (NBM), caudate, putamen, GP and NAc, according to the VTA atlas by Trutti *et al*.^20^ and the Allen human brain reference atlas^23^ (Supplementary Figs 1 and 2). Two analyses were conducted to evaluate the quality of the manually segmented VTA. In the first step, a second rater manually reconstructed a VTA ROI independently using the same manual reconstruction protocol as the first rater. The inter-rater agreement was then assessed using the dilated Dice score, a suitable reliability measure for small and complex shapes, such as the VTA, as previously described.^20^ The dilated Dice score indicated substantial overlap between the masks of the two raters (left = 0.851; right = 0.896).

In a second step, the manually reconstructed ROIs by the first rater were compared with a previously published probabilistic atlas of the VTA.^20^ The atlas was thresholded at 30% to eliminate low-probability voxels, then binarized. The comparison revealed agreement between the manual VTA masks and the VTA atlas, as evidenced by a dilated Dice score of 0.741 for the left VTA and 0.710 for the right VTA. All analyses were performed in ICBM 2009b Nonlinear Asymmetric space,^24^ using the ‘nighres.statistics.segmentation_statistics’ toolbox (v.1.4.0) from nighres^25^ in Python (v.3.8.12).

#### Brainstem

We used DSI Studio, a proprietary software package for dMRI developed by F.-C.Y.,^26^ to generate fibre-tracking results. We performed fibre tracking on a brainstem database of high-resolution diffusion-weighted imaging datasets to trace and reconstruct fibre bundles of the VTA using the VTA as an ROI.^22^ Imaging data were acquired for a total of 208 h using a total of 120 diffusion sampling directions with a b-value of 4000 s/mm^2^. The in-plane resolution was 0.2 mm and slice thickness 0.2 mm. The b-table was checked by an automatic quality-control routine to ensure its accuracy.^27^ The restricted diffusion was quantified using restricted diffusion imaging.^28^ Diffusion data were reconstructed using generalized *q*-sampling imaging^29^ with a diffusion sampling length ratio of 0.4. A deterministic fibre-tracking algorithm^30^ was used. A manually reconstructed ROI was placed at the VTA. The default quantitative anisotropy threshold was randomly selected within a range of 0.5–0.7. The angular threshold was randomly selected from 15° to 90°. The step size was 0.5 mm. Tracks with length <9 or >100 mm were discarded. The tracking process terminated when a total of 5000 seeds were reached.

#### Whole brain

We performed fibre tracking on the HCP-1065,^26^ a human population-averaged diffusion MRI template, which was generated from imaging data of 1065 subjects obtained from the HCP^31^ (WashU consortium) and individual data^32^ using DSI Studio. The diffusion data were acquired with b-values of 1000, 2000 and 3000 s/mm^2^. The number of diffusion sampling directions was 90, 90 and 90, respectively. The in-plane resolution and slice thickness were both 1.25 mm. To ensure accuracy, the b-table underwent scrutiny through an automatic quality-control routine.^27^ Subsequently, the diffusion data were reconstructed in the Montreal Neurological Institute (MNI) space using *q*-space diffeomorphic reconstruction^33^ to obtain the spin distribution function,^29^ with a diffusion sampling length ratio of 1.7. The process of quantifying restricted diffusion involved the application of restricted diffusion imaging techniques.^28^ The ROI used for fibre tracking was the VTA, and tract reconstruction was guided by our white matter dissection findings.

#### Microdissection-guided fibre-tract reconstruction

Tractography results derived from dMRI data necessitate validation using cadaveric data, as emphasized in prior literature.^34^ Fibre microdissection in cadaveric hemispheres constitutes a pivotal technique for studying the anatomy of fibre tracts in the human brain and is regarded as the ‘gold standard’ for validating dMRI findings.^34^ Accordingly, we qualitatively evaluated the detailed trajectory and connectivity of the generated tractography results in the context of our microdissection results, as previously described.^35,36^ Throughout the fibre microdissections, meticulous stepwise anatomical descriptions were recorded, accompanied by multiple photographs at each dissection stage, enabling evaluation of the tractography results and qualitative comparison with microdissection results on a ‘slice-by-slice’ basis. This evaluation was conducted in DSI Studio, wherein the detailed trajectory of the tract in 3D space was assessed relative to adjacent structures, such as cortical structures, white matter tracts, fissures, sulci and deep nuclei, along the route of the dMRI streamlines. We superimposed our fibre-tract reconstructions on sections of the 100 μm MGH^21^ and 50 μm Duke^22^ datasets, renowned for their histology-like resolution, facilitating a thorough examination of tract trajectory and comparison with histological findings. The comparisons between generated tractography results and microdissection findings were meticulously conducted on a ‘slice-by-slice’ basis, with direct comparisons facilitated by using dual screens for a more immediate and precise analysis. Streamlines deviating from the trajectory or connectivity observed in microdissections were deemed erroneous or false positives and consequently removed (Supplementary Figs 3 and 4). Our findings were documented visually through photographs, which were then juxtaposed with the tractography results under similar angles to facilitate a sequential sectional analysis and comparison. The correspondence between tractography and dissection underwent a qualitative evaluation through sequential sectional analysis by seven senior raters independently (V.A.C., L.B., F.-C.Y., A.H., M.K., A.M.L. and A.K.).

#### Connectivity-derived VTA parcellation

Our fibre-microdissection studies revealed a consistent topological organization pattern of VTA fibres within the VTA. Specifically, during our medial to lateral stepwise dissections, we observed fibres interconnecting different regions of the brain in a sequential order. Based on the specific pattern revealed by our microdissections, we hypothesized that the VTA is organized topologically and can be parcellated according to the location of the fibres within the VTA. Therefore, we isolated VTA fibre tracts according to their connectivity with different brain regions and outlined the space occupied by each tract within the VTA as previously described.^37^ DSI Studio enabled us to avoid manual outlining of each fibre tract using an automated fibre tract-to-ROI conversion function (Supplementary Fig. 5). After reconstructing each fibre tract using the HCP-1065 template, we trimmed the streamlines of the fibre tract outside the VTA to keep the streamlines of the fibre tract within the volume of the VTA ROI. We then converted the trimmed fibres into a ROI. We repeated this process for all fibre tracts and assigned different colours to each generated ROI. Finally, we overlayed all the generated ROIs and visualized them in 3D.

### Analysis of deep brain stimulation targets

To determine the potential clinical relevance of the tracts identified within the VTA, we carried out a strategic analysis of targeted hotspots and coordinates derived from prior research on the efficacy of DBS in the treatment of various neurological and psychiatric diseases. We conducted a comprehensive literature review to identify relevant studies published in the last 20 years. Search terms and inclusion criteria in terms of participants, interventions, comparisons, outcomes, studies (PICOS) are outlined in Supplementary Tables 1 and 2. Specifically, we focused on the ventral tegmentum and ventral diencephalon, which represent frequent targets in the management of obsessive–compulsive disorder (OCD) and major depressive disorder (MDD), cluster headache, aggression or self-injurious behaviour and Alzheimer’s disease. Anterior commissure–posterior commissure coordinates were converted into MNI coordinates as described previously^38^ and visualized as electrode trajectories in Lead-DBS.^39^ The defined coordinates represented the centre of the most distal contact along these trajectories. By following reported strategies and visualizations from the respective source publications, we then reconstructed lead trajectories.

To evaluate the spatial relationship between lead trajectories and the identified VTA tracts and to determine the extent of tract recruitment at each contact, we performed stimulation volume modelling. This involved estimating stimulation volumes in MNI space, using a modified version of the SimBio/Fieldtrip pipeline as introduced by Vorwerk *et al*.^40^ and implemented in Lead-DBS v.2.^41^ Through finite element modelling, we solved the Laplace equation within a discretized domain represented by a four-compartment mesh, including grey matter, white matter and the metallic and insulating parts of the electrode. Binary stimulation volumes were then generated by applying a heuristic E-field threshold of 0.2 V/mm, following the method proposed by Åström *et al*.^42^ This approach allowed us to model the dose–response relationship between stimulation amplitude and extent of fibre recruitment for each contact in a monopolar manner, using stimulation currents from 1.0 to 10.0 mA. For each stimulation volume, we calculated the percentage overlap with each identified tract of interest to identify the contact associated with maximal overage of each tract. The dose–response relationship at the optimal contact of each lead was then plotted for each tract.

## Results

### Fibre microdissections

**Figure 2:**
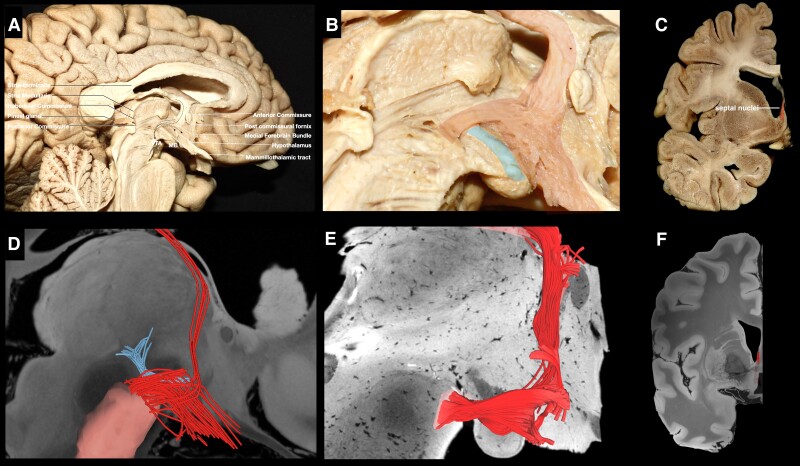
**VTA fibres running medial to the mammillothalamic tract.** (**A**) Medial view of a left hemisphere. The ependymal/subependymal layer has been removed to expose fibres running between the VTA, medial hypothalamus, fornix and septal region. (**B**) Magnified view of the area depicting the trajectory of the fibres highlighted in red and mammillothalamic fibres highlighted in pale blue. (**C**) Coronal section at the level of the termination points depicting septal nuclei highlighted in red. (**D**) Tractography depicting the VTA fibres in red and mammillothalamic tract in pale blue. (**E**) Brainstem *ex vivo* tractography depicting VTA fibres. (**F**) Coronal section depicting fibres of the VTA within the septal nuclei. VTA = ventral tegmental area.

**Figure 3:**
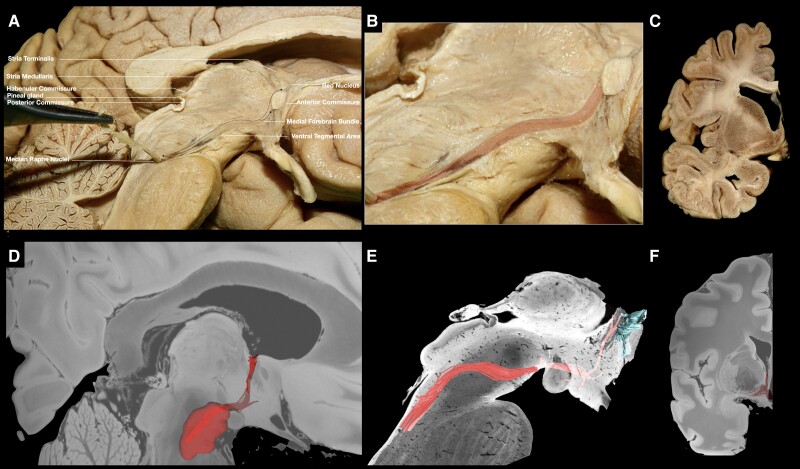
**VTA fibres implicating raphe nuclei, NBM and BNST.** (**A**) Medial view of a left hemisphere. The ventral mammillotegmental tract and mammillary body have been removed, exposing fibres running between the VTA, raphe nuclei, NBM and BNST. (**B**) Magnified view of the area, depicting the fibres highlighted in red. (**C**) Coronal section at the level of the termination point of VTA and stria terminalis fibres, depicting the BNST bounded by the anterior commissure inferiorly, the globus pallidus internus laterally, lateral ventricle medially and caudate nucleus superiorly. (**D**) Tractography showing VTA fibres in red. (**E**) Brainstem *ex vivo* tractography. (**F**) Coronal section, revealing tractography fibres within the BNST. BNST = bed nucleus of stria terminalis; NBM = nucleus basalis of Meynert; VTA = ventral tegmental area.

**Figure 4:**
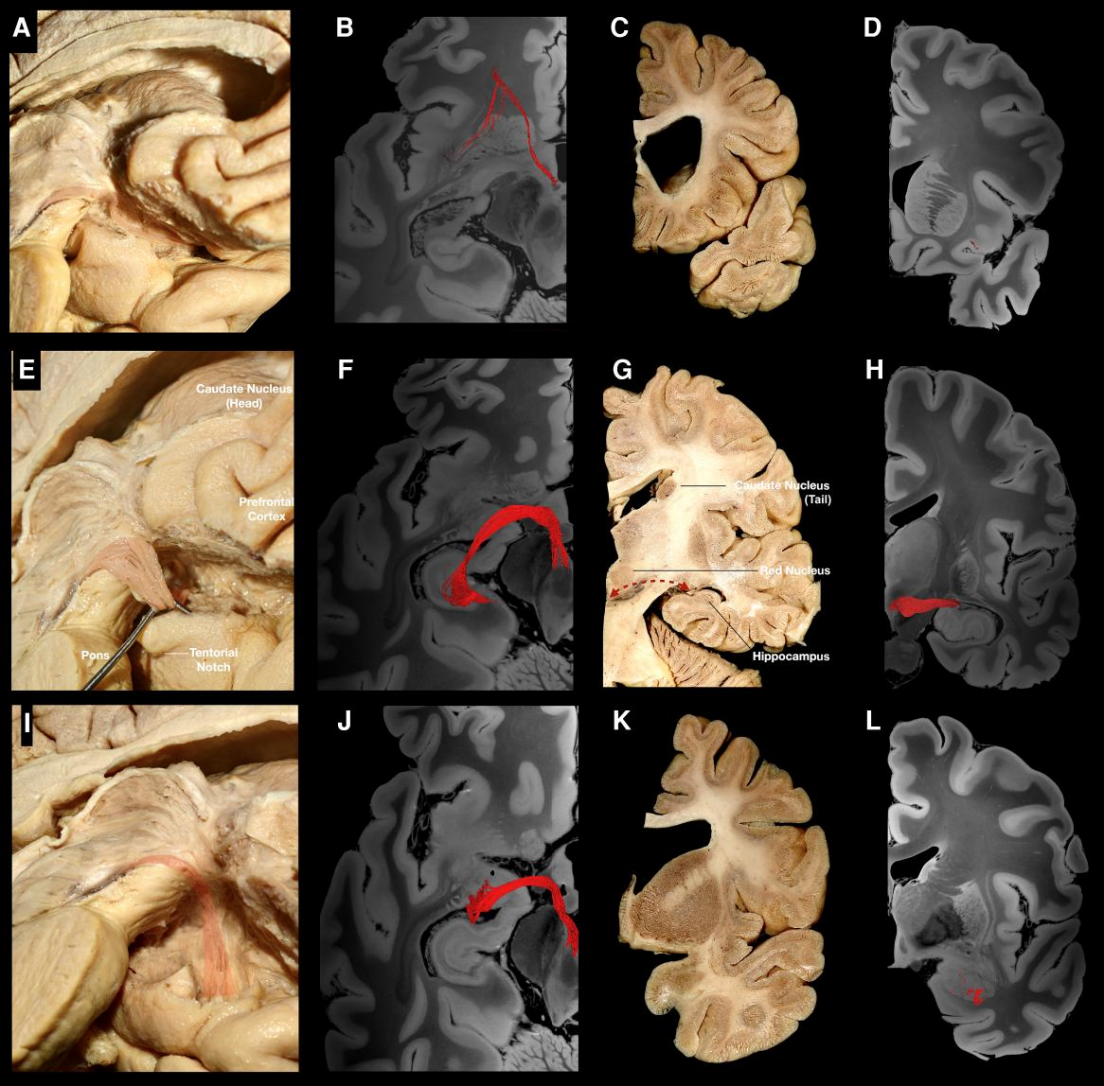
**Temporo-insular fibres.** (**A**) Medial view of a left hemisphere, showing fibres running between the VTA and insula highlighted in red. (**B**) Tractography, showing fibres running between the VTA and insula. (**C**) Coronal section at the level of the termination points, depicting the anterior insula. (**D**) Tractography, coronal section, revealing fibres within the insula. (**E**) Medial view of a left hemisphere following removal of insular fibres. Fibres running between the VTA and hippocampal region can be visualized arching laterally and posteriorly. (**F**) Tractography, showing fibres running between VTA and hippocampal area in red. (**G**) Coronal section at the level of the termination points, depicting the hippocampal area. (**H**) Tractography; coronal section, revealing fibres within the hippocampal area. (**I**) Medial view of a left hemisphere, showing fibres running between the VTA and amygdala/entorhinal cortex. (**J**) Tractography, showing the trajectory of the fibres between the amygdala/entorhinal cortex and VTA. (**K**) Coronal section of the contralateral hemisphere at the level of the termination points, depicting amygdala and entorhinal cortex. (**L**) Coronal section tractography, revealing VTA fibres within the amygdala. VTA = ventral tegmental area.

**Figure 5:**
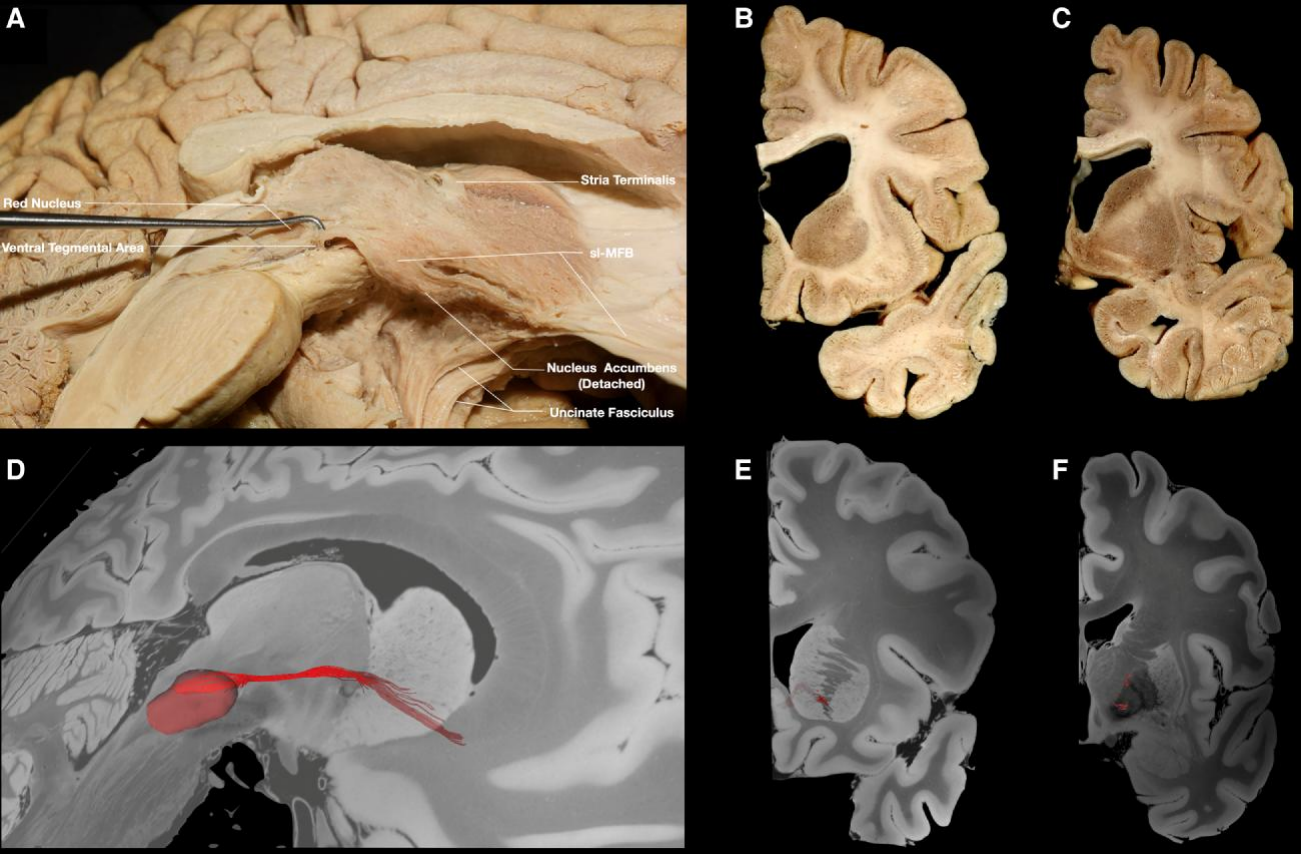
**GP and NAc.** (**A**) Medial view of a left hemisphere, showing fibres running between the VTA and basal ganglia region. (**B**) Coronal section at the level of the anterior termination points, showing the NAc. (**C**) Coronal section at the level of the posterior termination points, showing the GP. (**D**) Tractography, showing the trajectory of the fibres running between the VTA and NAc. (**E**) Tractography, coronal section, depicting VTA fibres within the NAc. (**F**) Tractography, coronal section, depicting VTA fibres within the GP. GP = globus pallidus; NAc = nucleus accumbens; slMFB = superolateral medial forebrain bundle; VTA = ventral tegmental area.

**Figure 6:**
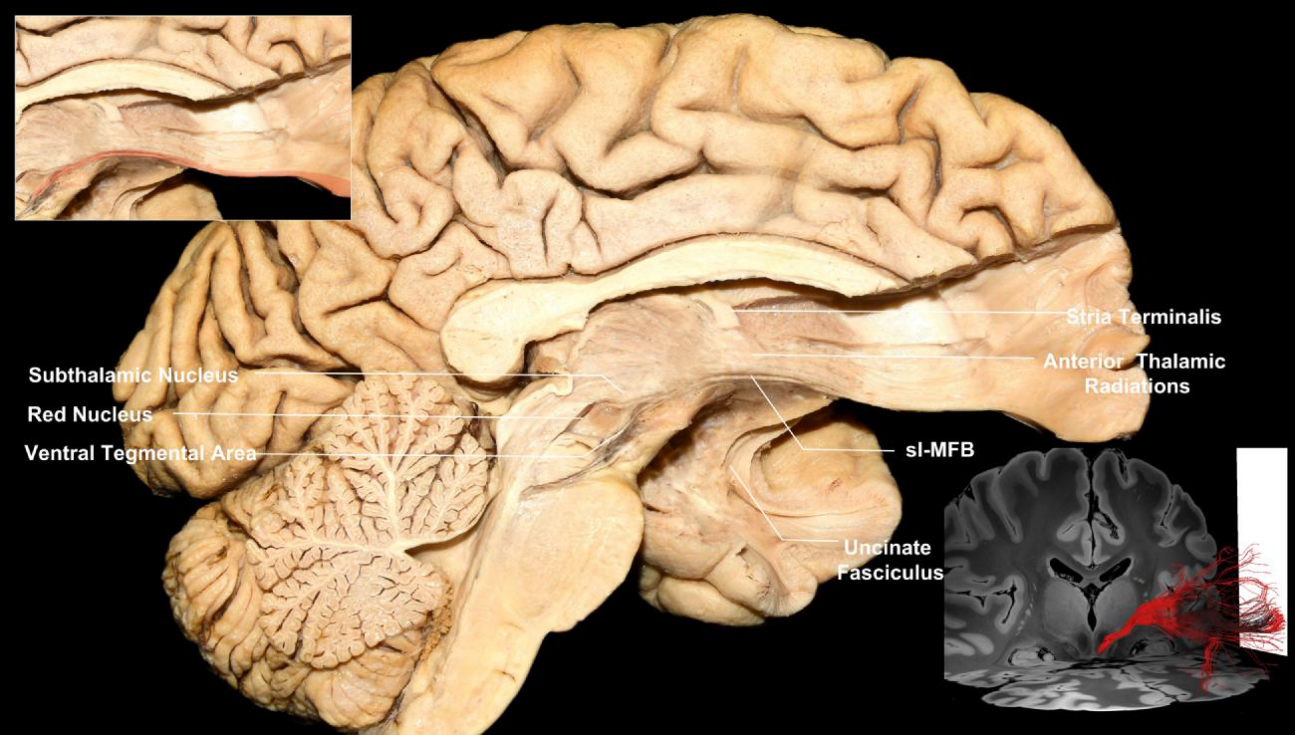
**VTA PFC fibres (BA10, BA11 and BA47/12).** Medial view of a left hemisphere, depicting fibres running within the ALIC between the VTA and BA10. *Top left inset*: magnification of the fibres implicating the VTA highlighted in red. *Bottom right inset*: tractography, showing fibres between the VTA and Brodmann areas 10, 11 and 47/12. ALIC = anterior limb of the internal capsule; BA = Brodmann area; PFC = prefrontal cortex; sl-MFB = superolateral medial forebrain bundle; VTA = ventral tegmental area.

Following the resection of the ependymal and subependymal layers lining the intraventricular surface of the third ventricle (Fig. 2A), fibres running between the VTA and basal forebrain were visualized medial to the mammillothalamic tract (MTT). These fibres fan out as they reach the post-commissural fornix. Some of the fibres curve superiorly either to blend with the post-commissural fornix or to continue towards the septal nuclei, while the inferior-most fibres were visualized to curve inferiorly towards the medial hypothalamus (Fig. 2). Following the dissection process laterally, the MTT can be visualized, and fibres running between the VTA and septal region are recorded lateral to the MTT (Supplementary Fig. 6). These fibres fan out anterior to the MTT. Some of the fibres curve superiorly and either blend with the post-commissural fornix or enter the region of substantia innominata/NBM, while the inferior-most fibres were visualized to curve inferiorly towards the lateral hypothalamus (Supplementary Fig. 6). Further stepwise dissection reveals the ventral mammillotegmental tract interconnecting the VTA with the mammillary body (Supplementary Fig. 7). Resection of the ventral mammillotegmental tract, MTT and fornix exposes a group of fibres interconnecting the VTA with the BNST and NBM (Fig. 3). Fibres interconnecting the VTA with the median raphe nuclei were also recorded during this step. Removal of these fibres exposes the hypothalamic nuclei and the paraterminal gyrus. At this point, no more fibres are observed to run in an anteroposterior direction, i.e. connecting areas of the basal forebrain with the VTA. Stepwise dissection of fibres from the VTA reveals fibres interconnecting the VTA with the anterior insula, hippocampus, dorsal dentate gyrus, amygdala and entorhinal cortex (Fig. 4). Following resection of the optic tract, hypothalamus, anterior commissure, ansa peduncularis, diagonal band, paraterminal gyrus, cingulate cortex, prefrontal cortex, underlying u-fibres, callosal radiations (forceps minor) and cingulum fibres, the shell of the ventral striatum is exposed along with fibres of the anterior limb of the internal capsule (ALIC) penetrating the shell of the ventral striatum, fibres of the inferior occipitofrontal fasciculus and fibres of the uncinate fascicle. Meticulous resection of the shell of the ventral striatum and cortical microdissection along the medial part of the head of caudate nucleus (CN) reveals a group of fibres running between the PFC and thalamus, along with fibres running between the VTA and basal ganglia, specifically the GP, putamen and NAc (Fig. 5). Resection of these fibres, along with further cortical microdissection of the CN and NAc, reveals the anterior thalamic radiations and fibres between the VTA and PFC contributing to the ALIC (Fig. 6). Resection of these fibres reveals more anterior thalamic radiations and fibres of the VTA terminating in Brodmann area (BA) 10, 11 and 47/12. At this level, further isolation of VTA fibres was not possible owing to the very dense criss-crossing pattern of fibres at the ALIC level.

### Tractography

We successfully reconstructed connections of the VTA through fibre tractography. Our extracted results were consistent with the structure, trajectory and connectivity of VTA fibres recorded during our microdissections. The VTA was connected to the raphe nuclei, hypothalamus, mammillary bodies, fornix septal nuclei, BNST, NBM, putamen, GP, insula, amygdala, hippocampus, dentate gyrus, NAc, entorhinal cortex, BA10, BA11 and BA12. Our connectivity-derived parcellation approach allowed us successfully to convert the fibre tracts within the VTA into ROIs and overlay them, thus creating a map representing the volume of each fibre tract within the VTA with a different colour (Supplementary Fig. 8). Fibres interconnecting the basal forebrain occupied the medial, anterior and superior regions of the VTA, fibres interconnecting the PFC, NAc and basal ganglia occupied lateral inferior and posterior regions of the VTA, and fibres interconnecting the insula and temporal lobe regions resided between them (Supplementary Fig. 8).

### Analysis of deep brain stimulation targets

**Figure 7:**
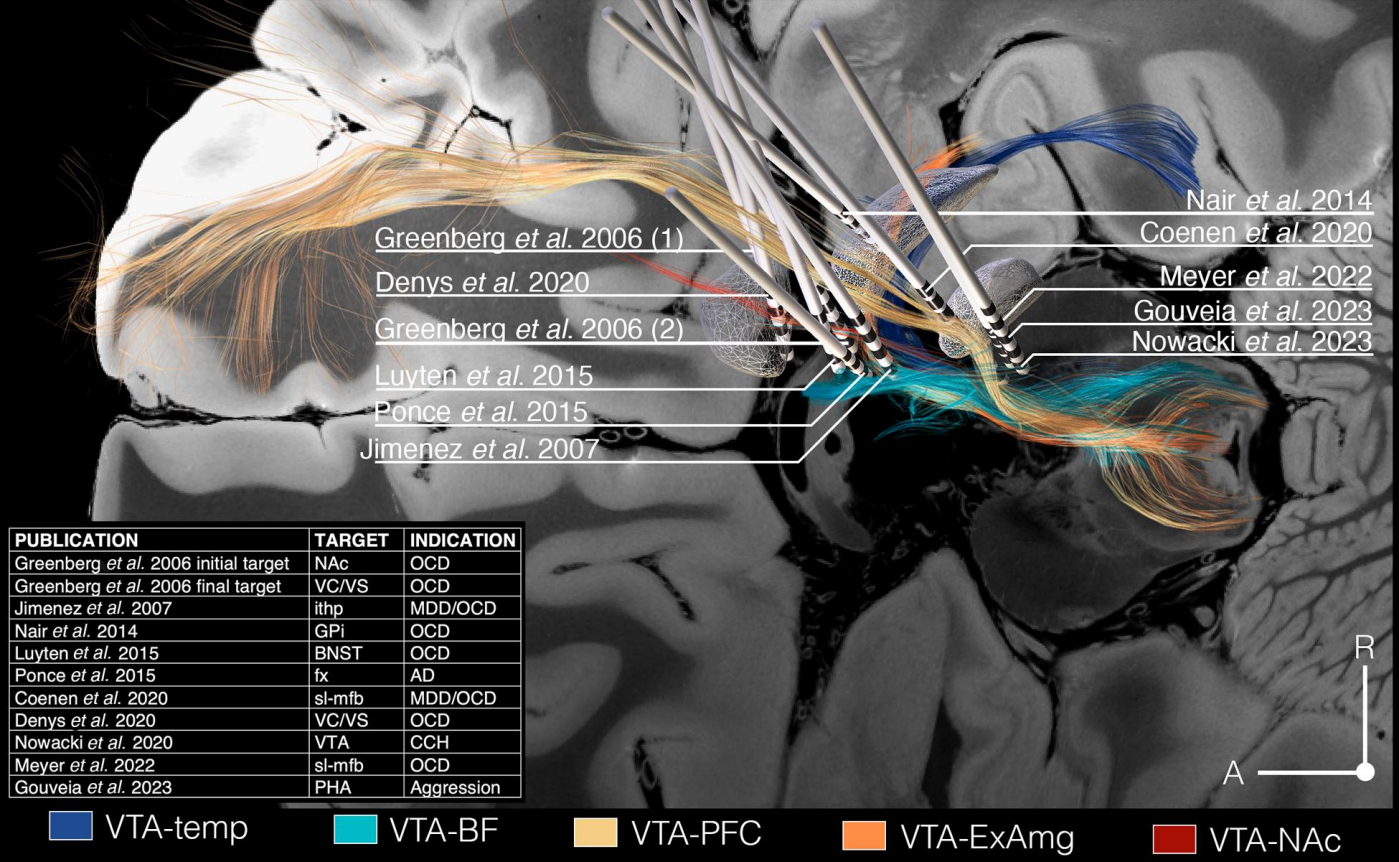
**Overview of VTA-related fibre tracts and DBS targets analysed.** Overview of DBS targets,^43-52^ indications and electrode placements relative to VTA tracts that we characterized. Fibre tracts are denoted as follows: VTA temp, fibre tracts interconnecting the VTA with the insula, hippocampus, dorsal dentate gyrus, amygdala and entorhinal cortex; VTA-BF, fibre tracts interconnecting VTA with hypothalamus, fornix (fx), septal region, nucleus basalis of Meynert, mammillary body, raphe nuclei and bed nucleus of stria terminalis; VTA-PFC, VTA-PFC fibres; VTA-ExAmg, fibre tracts interconnecting the VTA with the GP and extended amygdala; and VTA-NAc, fibre tracts interconnecting the VTA with the nucleus accumbens (NAc). DBS = deep brain stimulation; ithp = inferior thalamic peduncle; MDD = major depressive disorder; OCD = obsessive–compulsive disorder; PHA = posterior hypothalamus; VC/VS = ventral capsule/ventral striatum; VTA = ventral tegmental area.

**Figure 8:**
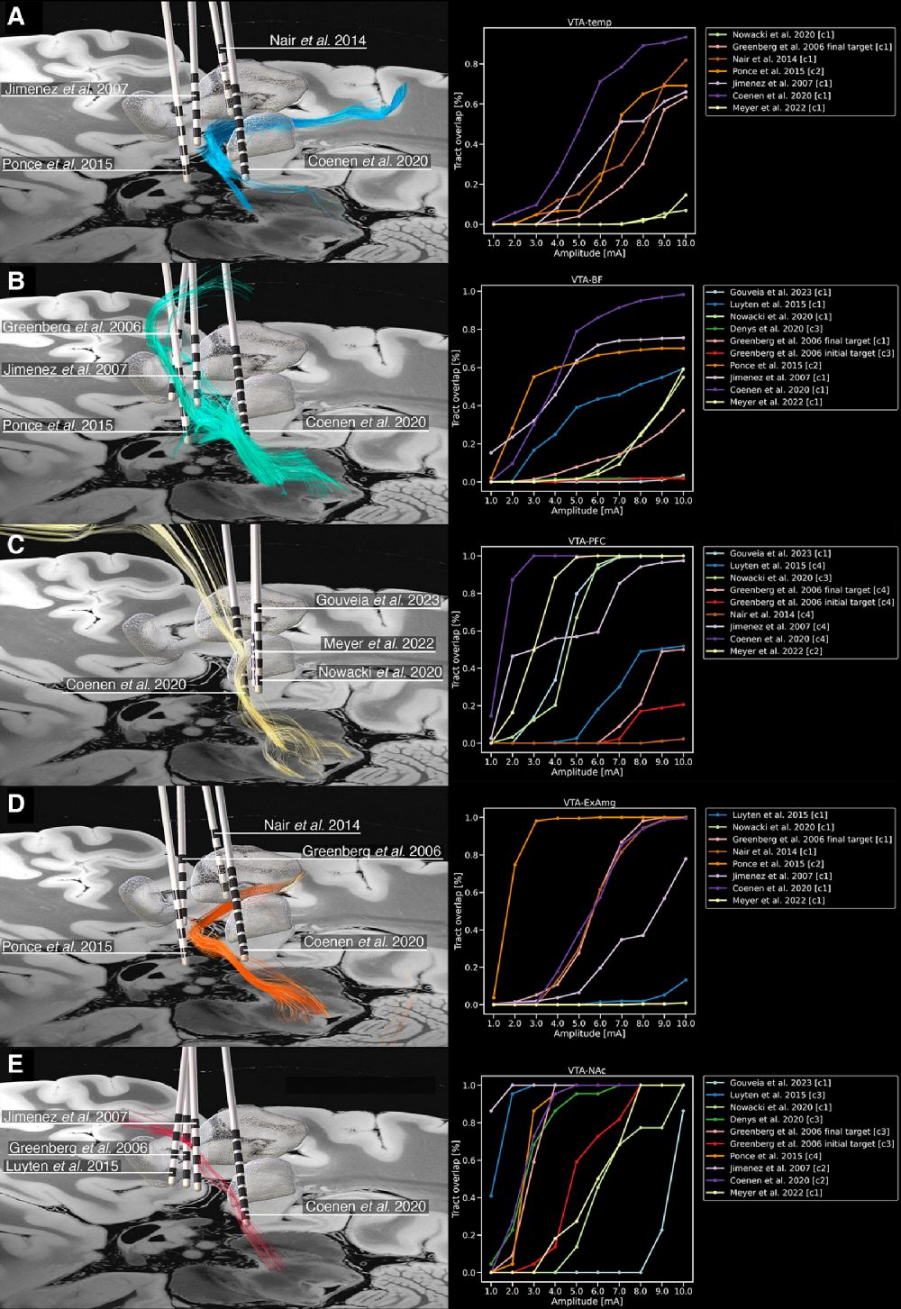
**Relationship between VTA tracts and established DBS targets.** (**A**) Fibre tracts interconnecting the VTA with the insula, hippocampus, dorsal dentate gyrus, amygdala and entorhinal cortex (VTA-temp) exhibited the highest ratio of percentage tract overlap to stimulation amplitude with ithp, slMFB, fornix and GPi DBS targets.^47-49,51^ (**B**) Fibre tracts interconnecting the VTA with the hypothalamus, fornix, septal region, nucleus basalis of Meynert, mammillary body, raphe nuclei and bed nucleus of stria terminalis (VTA-BF) exhibited the highest ratio of percentage tract overlap to stimulation amplitude with ithp, slMFB, fornix and NAc DBS targets.^44,47,49,51^ (**C**) Fibre tracts interconnecting the VTA with the BA10, BA11 and BA47/12 (VTA-PFC) exhibited the highest ratio of percentage tract overlap to stimulation amplitude with VTA, slMFB and posterior hypothalamus DBS targets.^45,50-52^ (**D**) Fibre tracts interconnecting the VTA with the GP and extended amygdala (VTA-ExAmg) exhibited the highest ratio of percentage tract overlap to stimulation amplitude with fornix, GPi and NAc DBS targets.^44,48,49,51^ (**E**) Fibre tracts interconnecting the VTA with the NAc (VTA-NAc) exhibited the highest ratio of percentage tract overlap to stimulation amplitude with BNST, NAc, ithp and slMFB DBS targets.^44,46,47,51^ BNST = bed nucleus of stria terminalis; DBS = deep brain stimulation; GPi = globus pallidus internus; ithp = inferior thalamic peduncle; NAc = nucleus accumbens; slMFB = superolateral medial forebrain bundle; VTA = ventral tegmental area.

We identified 10 representative clinical studies that met our inclusion criteria.^43-52^ These studies provided target coordinates, electrode trajectories or hotspots either in MNI space or relative to the anterior commissure–posterior commissure line. Moreover, the selected studies featured cohorts comprising ≥10 patients to ensure an adequate sample size. We successfully reconstructed electrode trajectories and our characterized VTA tracts in MNI space by using our fibre-tractography data and previously reported DBS data (Fig. 7). Reconstruction of the lead trajectories and characterized tracts allowed us to evaluate the dose–response relationship between stimulation amplitude and fibre recruitment (Fig. 8).

In terms of stimulation outcomes, all identified tracts were modulated by leads implanted within the ventral tegmentum/ventral diencephalon. Among these, the VTA-BF, VTA-NAc and VTA-PFC tracts showed the most extensive coverage. Notably, the VTA-NAc demonstrated the most efficient recruitment, characterized by the highest ratio of percentage tract overlap to stimulation amplitude. Investigation of lead trajectories revealed that the targets determined by Nowacki *et al*.^52^ (VTA stimulation in chronic cluster headache), Meyer *et al*.^45^ (slMFB stimulation in OCD), Jiménez *et al*.^47^ [stimulation of the inferior thalamic peduncle (ithp) in OCD], Greenberg *et al*.^44^ (NAc DBS in OCD), and Coenen *et al*.^51^ (slMFB DBS in MDD and OCD) exhibited the most comprehensive coverage, recruiting streamlines associated with all investigated fibre tracts. The targeting approach defined by Nowacki *et al*.^52^ yielded the most potent recruitment of VTA tracts among the evaluated implantations sited, indicating a significant interaction of electrical stimulation with the dopamine system. This was followed by lead trajectories reconstructed from the studies of Meyer *et al*.^45^ and Jiménez *et al*.,^47^ underscoring the recruitment of common fibre tracts across variable implantation sites.

## Discussion

Using both cadaveric microdissection and *in vivo* fibre-tractography approaches, the VTA was consistently found to be interconnected with the raphe nuclei, hypothalamus, mammillary bodies, fornix, septal nuclei, BNST, NBM, the caudate, the putamen, GP, extended amygdala, insula, amygdala, hippocampus, dorsal dentate gyrus, NAc, entorhinal cortex and PFC. The VTA is an integral hub of an extended network including circuits that facilitate memory consolidation^53^ and global cognitive recovery following stroke^54^ and are implicated in the pathophysiology of Parkinson’s disease, MDD, post-traumatic stress disorder, schizophrenia, neurocognitive symptoms in epilepsy and affective behaviours.^55-59^ Our VTA parcellation informs personalized DBS approaches, aiming at symptom relief and prevention of side effects by targeting or avoiding specific connections. DBS of the VTA region has been performed for cluster headaches, OCD, MDD, aggressive behaviour, atypical facial pain and anorexia nervosa.^8^ Preclinical studies have proposed the VTA as a potential DBS target for seizure control.^60,61^ Our findings provide a structural substrate apprising DBS in the VTA region based on direct human structural data. By reconstructing reported lead trajectories, we were able to evaluate the relationship between patient-specific DBS targets and the identified VTA tracts. Although clinical conclusions cannot be drawn, this qualitative assessment allowed us to appreciate that the tracts we characterized are modulated during DBS for OCD, MDD, Alzheimer’s disease, aggression and cluster headaches.

Tractography studies can reveal altered brain connectivity when compared with normal circuits and are the principal method for the identification of neural circuits implicated in DBS targets.^62,63^ However, tractography is prone to false positives, and results should be validated through cadaveric studies.^34^ Our fibre-microdissection-guided results contribute to the exploration of VTA-related neural circuits and altered connectivity by aiding in the differentiation of potential false-positive connections. Moreover, our findings can inform dMRI studies and help to guide tractography-guided DBS. This approach is key to identify both treatment-related and side effect-related circuits to enhance preoperative DBS planning and improve postoperative outcomes.^64^ Normative connectomes constitute the main source for characterizing these circuits; nevertheless, they are prone to false-positive connections.^9,17,34^ Structural knowledge of the normal circuitry can facilitate individualized target selection according to individual symptoms and imaging characteristics.^64^ These circuits could be leveraged to help tailor DBS in patients through open-loop and closed-loop approaches. Symptom- and biomarker-specific approaches can be implemented through differential tractography for the identification of fibre tracts that are altered in patients and targeting of fibre tracts that are related to individual symptoms.^63^ Accordingly, knowledge of VTA-related circuits could help to optimize detailed lead placement in closed-loop approaches and the selection of stimulation parameters that could exploit symptom-specific networks. Our DBS hotspot analysis results allowed us to assess the anatomical relationship between VTA tracts and established DBS targets, suggesting that this network is indeed modulated during DBS for OCD, MDD, Alzheimer’s disease, cluster headaches and aggressive behaviours. Recent intracranial recording studies have sought to identify neurophysiological characteristics in patients with psychiatric disorders.^65^ Our findings can inform potential targets for intracranial recordings and facilitate sampling of relevant circuits.

### Dorsal raphe nucleus

Efferent fibres of the raphe nuclei synapsing within the VTA comprise the main afferent neuron populations synapsing with GABA- and glutamate-releasing VTA neurons and are involved in aversive stimuli-related outcomes.^66^ The role of serotonin and the raphe nuclei has been established in the pathophysiology of MDD.^67,68^ The therapeutic effects of VTA DBS in patients with MDD have been attributed to fibres interconnecting the VTA, NAc and PFC.^10^ Here, we provide human evidence demonstrating that stimulation of the VTA implicates a direct pathway between the VTA and raphe nuclei that might facilitate the therapeutic effects of DBS for MDD. Preclinical studies have demonstrated that VTA neurons modulate the activity of dorsal raphe nucleus serotonergic neurons.^69^ This direct connection is likely to explain the increase of serotonin levels measured within the PFC following MFB self-stimulation in a preclinical model of induced depression.^70^ Given that raphe nuclei are involved in the pathophysiology of Alzheimer’s disease, this pathway might have further clinical implications in the diagnosis and treatment of patients with Alzheimer’s disease.^71^ The strong correlation of Parkinson’s disease with raphe nuclei^72,73^ and the robust degeneration localized within regions we found connected to the VTA, namely the ventral striatum, CN, GP, insula and PFC,^74^ suggests that the VTA might present a promising stimulation target for Parkinson’s disease. The distinct connections we identified might facilitate early diagnosis and symptom-specific phenotyping of Parkinson’s disease.

### Hypothalamus and basal forebrain

Projections of the lateral hypothalamus to the VTA have been studied comprehensively for over a half-century owing to their significance in appetitive, reward and goal-directed behaviour.^75-77^ Notably, this pathway interconnecting the hypothalamus and VTA has been described as the MFB in animals and humans.^78-81^ According to animal studies, this pathway carries VTA-originating axons that co-release glutamate and GABA, in addition to GABAergic hypothalamic neurons.^66^ These connections are very likely to contribute to autonomic cardiac side effects related to DBS of the VTA region.^82^ Although stimulation of the hypothalamic region has been suggested to modulate the slMFB,^83^ the effects of hypothalamic modulation through VTA stimulation have not been assessed thoroughly. We found that VTA fibres terminating in the hypothalamus would consistently occupy medial and superior regions within the VTA (Fig. 8). Electrode placement in more posterolateral locations within the VTA might minimize stimulation of these fibres; therefore, reducing autonomic-related side effects. Efferents of the lateral hypothalamus to the VTA inhibit GABA-releasing VTA neurons, resulting in increased dopaminergic NAc activity.^84^ Animal studies have shown that hypothalamus–pituitary–adrenal axis alterations related to chronic stress modulate dopamine transmission through the VTA.^85^ Aberrant stress responses are a common component of OCD and MDD, conditions which have been treated successfully with VTA DBS.^8^ Recent preclinical studies suggest that neuromodulation of VTA neurons involved in hypothalamic circuitry might provide treatment of anxiety disorders.^86^ Animal studies report connections of the VTA with the medial hypothalamus via the MFB.^87,88^ The neural circuit involving the VTA and medial hypothalamus has further implications in the regulation of progesterone and adrenocorticotrophic hormone^89,90^ and plays a key role in aggression.^91^ VTA DBS has been performed successfully for aggressiveness.^8^ Our findings indicate that the VTA is connected to the medial hypothalamus; therefore, these fibre tracts might mitigate the therapeutic effects of VTA DBS for aggressiveness.

Although animal studies have characterized two parallel pathways interconnecting the mammillary body to the dorsal and ventral tegmentum,^92^ the connectivity of the mammillotegmental tract in humans has not been clarified. Our findings demonstrate that fibres interconnect the VTA with the mammillary body through distinct a ventral mammillotegmental tract, which is separated from the mammillotegmental tract by the red nucleus and adjacent structures (Supplementary Fig. 4). In animals, the mammillary body and VTA are interconnected through the MFB.^15,93,94^ This ventral pathway is essential in supporting memory.^95^ Hence, the ventral mammillotegmental tract might be implicated in Korsakoff’s syndrome and other dementias as a biomarker for early diagnosis or prediction for DBS response. The pathway involving the VTA and fornix has important implications in memory and modulates the activity of neurons interconnecting the VTA and PFC.^96,97^ The therapeutic effects of fornix DBS for Alzheimer’s disease have been attributed, in part, to indirect connections of the fornix to the VTA through the connectivity of the mammillary body and VTA.^98^ Here, we provide data showing the direct connectivity of the fornix and VTA, and demonstrate that the VTA is directly connected to areas that have been targeted successfully for Alzheimer’s disease with DBS, such as the entorhinal cortex, ventral striatum and NBM.^99^ Thus, the VTA might offer a more potent DBS target modulating multiple regions involved in the pathophysiology of various dementias.

The bidirectional pathway interconnecting septal nuclei with the VTA modulates the activity of both the VTA and septal nuclei.^100^ In the rat, this pathway contains a population of orexin neurons.^101^ More recent studies have shown that glutamate-releasing VTA efferents to the septal nuclei modulate anxiety behaviours.^102^ Moreover, dopamine-releasing VTA efferents to the septal nuclei have an inhibitory effect on GABA neurons and modulate aggressive behaviours.^103^ Modulation of the connections we characterized between VTA and septal nuclei might mitigate aggressive behaviours following VTA DBS.^8^ The pathway interconnecting the VTA and NBM carries cholinergic afferents to dopaminergic VTA neurons, afferents to GABAergic VTA neurons, and afferents to glutamatergic NBM neurons, involving motor, stress and depressive behaviours.^104-106^ Although the VTA and the NBM exhibit abnormal function in Parkinson’s disease and Alzheimer’s disease, this pathway has not been identified in the human brain.^107,108^ Therefore, further studies should elucidate its role as a potential DBS target or biomarker for early diagnosis of Parkinson’s disease and Alzheimer’s disease. Fibres interconnecting the VTA with BNST carry GABAergic BNST efferent neurons and are modulated by chronic stress, chronic pain and alcohol withdrawl.^109-111^ Additionally, the VTA-BNST circuit regulates reward, anxiety, punishment and maladaptive behaviours.^112^ The BNST has been used successfully as a DBS target for OCD and MDD.^9,113^ This raises the question of whether therapeutic effects of BNST and VTA DBS for affective disorders are both facilitated through the pathway we identified. Our findings can help the symptom/side-effect characterization of this tract.

### Medial temporal lobe and insula

We found robust connections in the hippocampus/dorsal dentate gyrus, amygdala and entorhinal cortex. Clinical data demonstrate elevated mesial temporal lobe metabolism in treatment-resistant depression patients who have undergone MFB DBS in the VTA region, thereby supporting the connectivity of these regions.^114^ The structural connectivity between the hippocampus and VTA has been reported recently in humans; nevertheless, the authors used only indirect dMRI data to study this connection and did not report data regarding the anatomy, trajectory or directionality of the tract.^115^ VTA neurons connecting the entorhinal cortex encode for memory and learning.^116^ The connectivity between the VTA and hippocampus supports memory and plays a major role in the pathophysiology of Alzheimer’s disease.^117,118^ Fibre tracts interconnecting the amygdala and VTA regulate anxiety-related behaviours,^119^ underscoring the potential implications of the tract we report as an efficacious stimulation target and biomarker for early diagnosis of anxiety-related disorders.

The insula supports sensory, emotional and higher-order processing.^120^ It is implicated in psychiatric disorders and has been suggested as a promising brain stimulation target for addiction.^121^ Dopamine and 5-hydroxytryptamine type 1a receptors are overexpressed in the insula.^122^ Optogenetic stimulation of the VTA induces dopamine release in the insula and modulates memory.^123^ Administration of renin–angiotensin system-targeting drugs in humans attenuates the functional connectivity between the VTA and insula in response to social punishment, suggesting that renin–angiotensin system-targeting drugs reduce the aversive emotional effect of social punishment feedback on the insula.^124^ The role of the insula in the pathophysiology of addiction and its relationship to the VTA is supported further by a recent study reporting lower fractional anisotropy values in insula-NAc and VTA-NAc fibres in long-term heroin abstinence subjects.^125^ The functional connectivity between the VTA and insula plays a key role in a network implicating the PFC, anterior insula, amygdala and VTA, which is activated during self-efficacy belief formation.^126^ Maladaptive self-efficacy beliefs leading to feelings of worthlessness constitute a phenotypic hallmark in MDD.^127^ MDD patients exhibit attenuated functional connectivity between the VTA and insula during reward anticipation compared with healthy individuals.^128^ Chronic alcohol exposure significantly alters the structural and functional connectivity between insula and VTA.^129^ In a recent study investigating neural circuits implicated in prosocial behaviour, the VTA and insula were involved in making choices for oneself and not in choices that benefit others, underscoring the unique role of this pathway in higher-order function.^130^ As such, abnormal connectivity between the insula and VTA might be related to self-belief and social dysfunction symptoms of psychiatric disease, and VTA DBS targeting the pathway connecting the VTA to the insula might specifically attenuate these symptoms.

### Globus pallidus and extended amygdala

GP DBS has been reported to alleviate symptoms for patients with OCD.^48,131^ Our analysis indicated that four different DBS targets (slMFB, ithp, BNST and GP internus) successfully used for the treatment of OCD modulate the same set of fibre tracts interconnecting the VTA with the GP and extended amygdala (Fig. 8). This pathway was recently shown to be implicated in anxiety associated with cocaine withdrawal.^132^ The extended amygdala plays a significant role in processes related to fear, anxiety and addiction^133^ and receives inputs from the rostral zona incerta,^134^ which was recently suggested as a potential DBS target for OCD. DBS directly to the extended amygdala has improved emotional, social and cognitive symptoms of autism and self-injury.^135^ Future studies on patients with Parkinson’s disease should explore whether VTA DBS or tractography-guided DBS of the fibres interconnecting the VTA with the GP might offer alleviation of cognitive and affective symptoms with fewer side effects.

### Nucleus accumbens and prefrontal cortex

Connections between the VTA and NAc are intricately related to the VTA–hypothalamus circuit, a network which is modulated by leptin and supports reward feeding behaviours.^136^ Moreover, this connections plays a key role in chronic emotional stress and anxiety-related behaviours.^137^ In humans, the fibre tract interconnecting the VTA with the NAc has been associated with impulsivity.^138^ VTA efferents to the PFC are modulated by chronic stress, which induces different structural and functional sex-dependent changes.^139^

The connectivity between the VTA and PFC is facilitated by a fibre tract that runs in the ALIC, replicated by many previous imaging studies, that has been named the slMFB.^140^ The slMFB has been a matter of controversy regarding its connectivity and its name.^10^ Arguments regarding inappropriate nomencalcture are based on animal studies that refer to the internal capsule and MFB as anatomically distinct structures.^9^ Notably, animal studies have reported a tight anatomical relationship or overlap between the MFB and internal capsule.^141-143^ However, our study does not aim to resolve any nomenclature-related controversies. Our results regarding the trajectory and structure of the tract connecting the PFC with VTA are aligned with previous descriptions of the slMFB.^140^ Our study defined a detailed 3D reconstruction of the tract, supporting previous imaging data through white matter microdissection evidence, for the first time, and validating our microdissection results through a two-ROI fibre-tractography approach. PFC glutamatergic efferents running within the slMFB have recently been reported in a large non-human primate study.^144^ DBS of the slMFB has been associated with high treatment response rates in patients with MDD and OCD.^145^ Structural alterations of the slMFB have been associated with alcohol use disorder, bipolar disorder, MDD and psychosis disorders.^146-148^

### Limitations

Despite the thorough investigation conducted in this study, the inherent limitations of both Klingler’s technique and tractography should be always taken into consideration. Both methodologies are susceptible to yielding false negatives, particularly in regions characterized by high white matter density and an abundance of kissing and crossing fibres.^18,36^ Tractography is prone to false-positive results and should be validated by histological data.^149^ However, the comparison between histological and imaging data can only be achieved qualitatively. Despite these challenges, Klingler’s technique remains the sole available approach for examining fibre tracts directly within the human brain.^34^ Future studies should focus on the technological developments that will allow the quantitative comparison between histological and imaging data in a common space. Moreover, our connectivity-derived parcellation of the VTA is based on delineating the volumes occupied by the different fibre tracts within the VTA using an averaged template created from 1065 healthy subjects. Consequently, our findings can only suggest a generalized topology template of the fibre-tract organization within the VTA. However, it is crucial to consider the individual imaging characteristics of each patient meticulously in the clinical setting and during tractography-guided neuromodulation procedures. Furthermore, our study encountered challenges in discerning fibres within the internal capsule and potentially failed to capture connections with other regions of the human brain. Additionally, the distinction of fibres connecting the VTA with globus pallidus internus, globus pallidus externus and external amygdala proved elusive. Moreover, it is important to note that the fibre-microdissection technique used herein is unable to distinguish the origin versus termination of a tract.

## Conclusions

Our microdissection and tractography findings converge, indicating that the VTA is connected to the raphe nuclei, hypothalamus, mammillary bodies, fornix, septal nuclei, NBST, NBM, caudate, putamen, GP, insula, amygdala, dorsal hippocampus/dentate gyrus, NAc, entorhinal cortex, BA10, BA11 and BA12. The VTA is integral to limbic, striatal, basal forebrain and prefrontal circuits. Our findings demonstrate that the VTA is an integral hub of an extended network involving the serotonergic pontine nuclei, limbic system, basal forebrain, basal ganglia and PFC, which modulates action, reward, memory, drug-seeking, addiction, aggression and anxiety-related behaviours. Accordingly, the VTA offers a promising sophisticated DBS target for neuropsychiatric disorders such as Korsakoff syndrome, Parkinson’s disease and Alzheimer’s disease and should be assessed through future preclinical studies. Connections of the VTA exhibit a topographical organization within the VTA according to their connectivity with: (i) the hypothalamus and basal forebrain; (ii) insula and temporal lobe; and (iii) NAc and PFC.

## Supplementary Material

awae173_Supplementary_Data

## Data Availability

Imaging data used in this study are publicly available through the human connectome project (https://www.humanconnectome.org/) and DSI studio (https://dsi-studio.labsolver.org/). Human cadaver data are not publicly available owing to conflicts with privacy reasons.
