# Supplementary material for: Establishing connectivity through microdissections of midbrain stimulation-related neural circuits
Source: Brain. 2024 May 29;147(9):3083–98. doi: 10.1093/brain/awae173 (PMC11370807; doi:10.1093/brain/awae173)
Supplement: awae173_Supplementary_Data [file awae173_supplementary_data.pdf]

Table S1.

Search term and conditions

**Search terms**	(deep brain stimulation) AND midbrain<br>(deep brain stimulation) AND diencephalon<br>(deep brain stimulation) AND fornix<br>(deep brain stimulation) AND thalamus
**Dates**	2004-Present

Table S2.

Inclusion criteria in terms of PICOS

**Participants**	All patients of any age and gender
**Interventions**	Deep Brain Simulation of the midbrain or diencephalon.
**Comparisons**	n/a
**Outcomes**	1. Clinical presentation<br>2. Etiology<br>3. Clinical diagnosis<br>4. DBS target and parameters (target coordinates, electrode trajectories, or hotspots either in MNI space or relative to the AC-PC line)
**Study design**	observational studies and clinical trials

Abbreviations: PICOS= Participants, Interventions, Comparisons, Outcomes, Study design

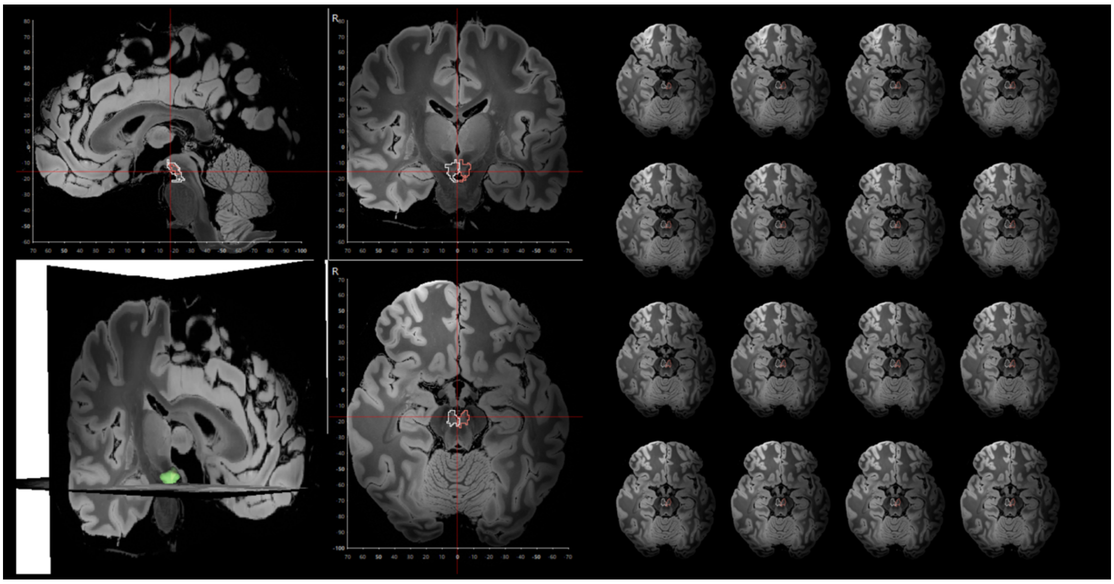

Figure S1. Manual reconstruction of the Ventral Tegmental Area ROI on the 100µm MGH dataset.

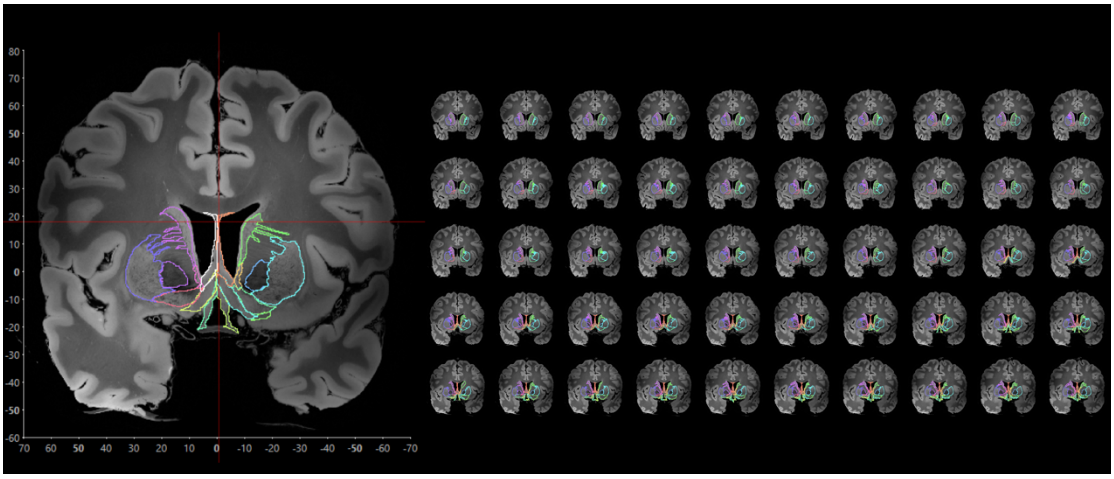

Figure S2. Manual ROI reconstruction of areas connected to the Ventral Tegmental Area on the 100µm MGH dataset.

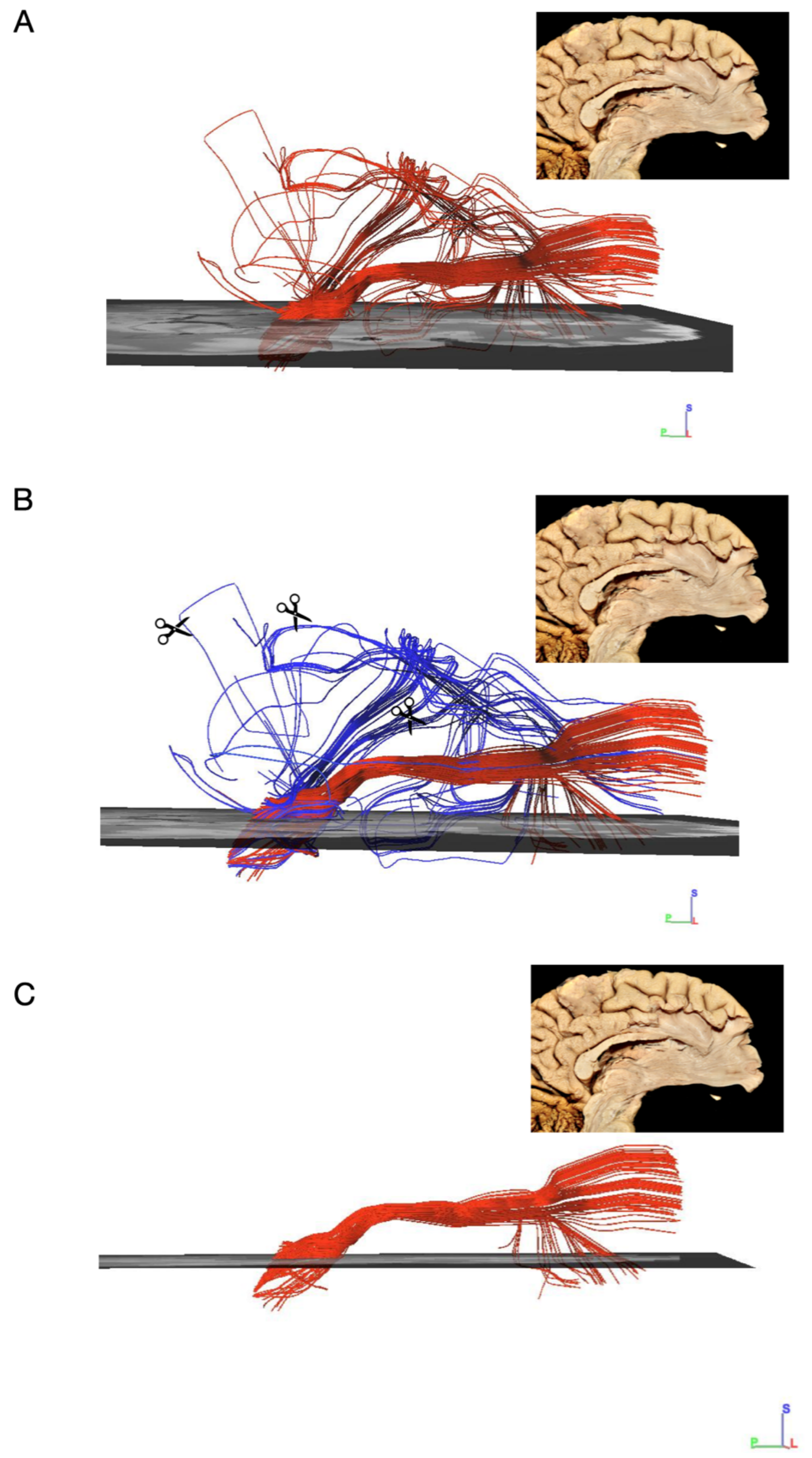

Figure S3. Microdissection guided fiber tract reconstruction. Example demonstrating stepwise exclusion of tracts that were not confirmed histologically. After initial fiber tracking results are generated, the results are superimposed on high resolution datasets (A) and the detailed trajectory of the dMRI streamlines is meticulously assessed in the context of the histological results. Streamlines deviating from the trajectory observed in microdissections (depticted in blue) are removed (B, C).

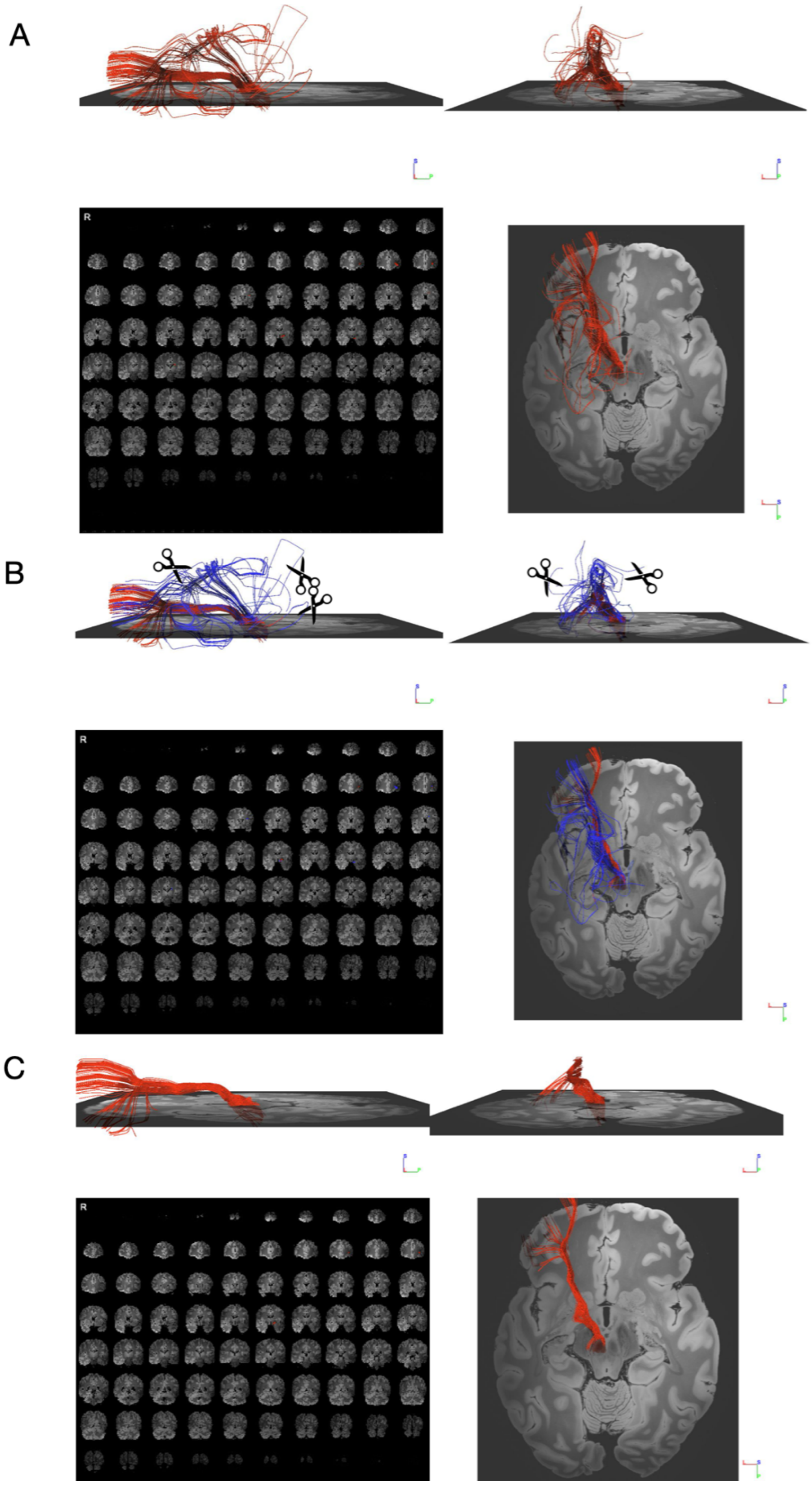

Figure S4. Microdissection guided fiber tract reconstruction (continued). The comparisons between initial tractography generated results and microdissection findings were meticulously conducted by assessing the trajectory of the streamlines in 3D from multiple angles and their relationship with adjacent structures on a “slice-by-slice” basis (A). Streamlines deviating from the trajectory observed in microdissections (depticted in blue) are removed (B, C).

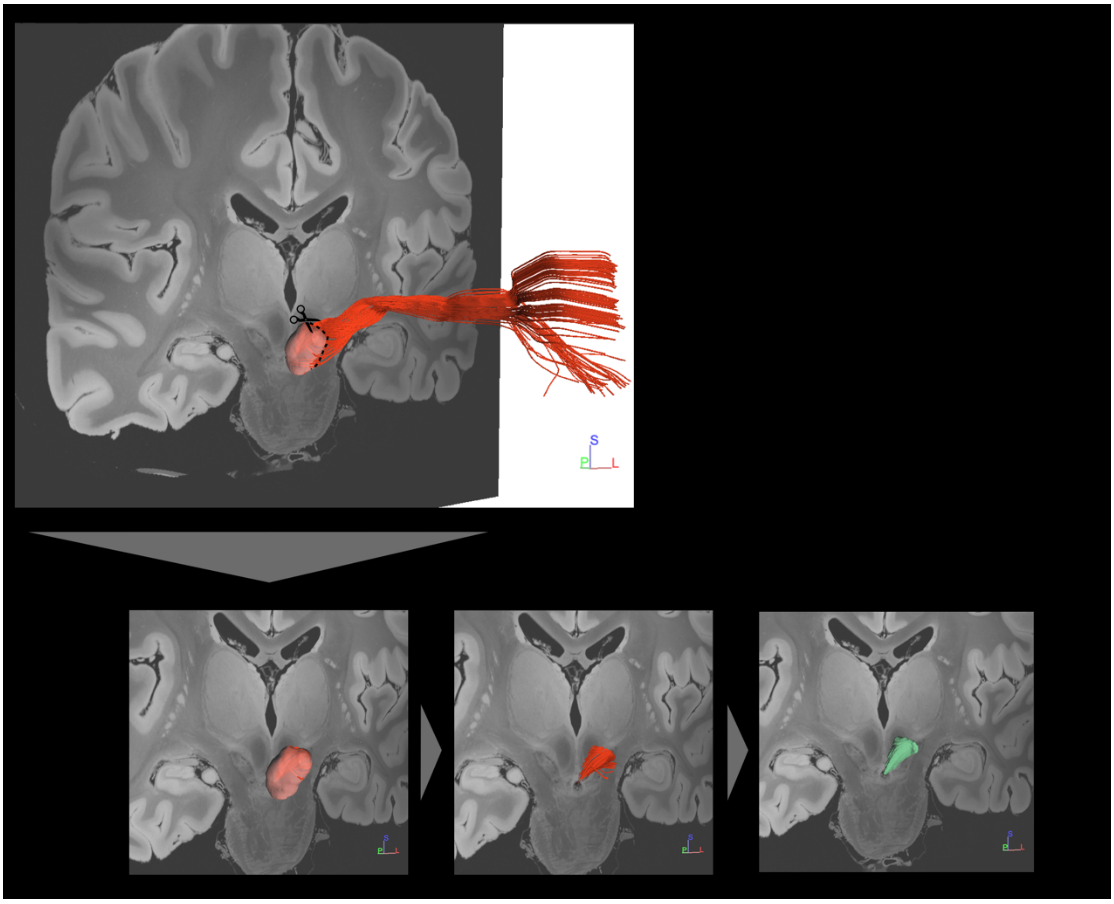

Figure S5. Example of conversion of tracts to ROI to delineate the occupying volume of the tract within the ventral tegmental area. After reconstruction of the tract, the streamlines were trimmed outside the VTA to keep the fiber tract within the volume of the VTA ROI. The trimmed fibers were then converted into a ROI.

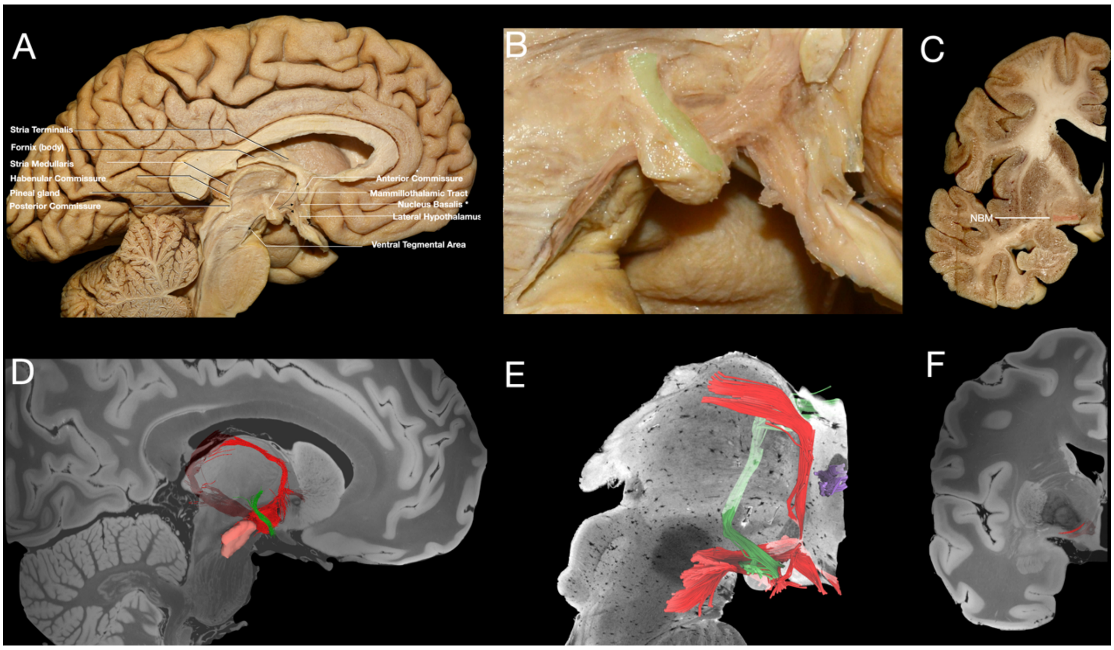

Figure S6. VTA fibers running lateral to the mammillothalamic tract (MTT). **A.** Medial view of a left hemisphere showing fibers running lateral to the MTT between the VTA and lateral hypothalamus, fornix, and NBM. **B.** Magnified view of the area depicting the directionality of the fibers highlighted in red. **C.** Coronal section at the level of the termination points depicting the NBM. **D.** Tractography depicting the VTA fibers in red and MTT in green. **E.** Brainstem ex-vivo tractography depicting VTA fibers in red, MTT in green and AC in purple. **F**. Tractography, coronal section depicting fibers within the NBM. AC, anterior commissure; MMT, mammillothalamic tract; NBM, nucleus basalis of Meynert.

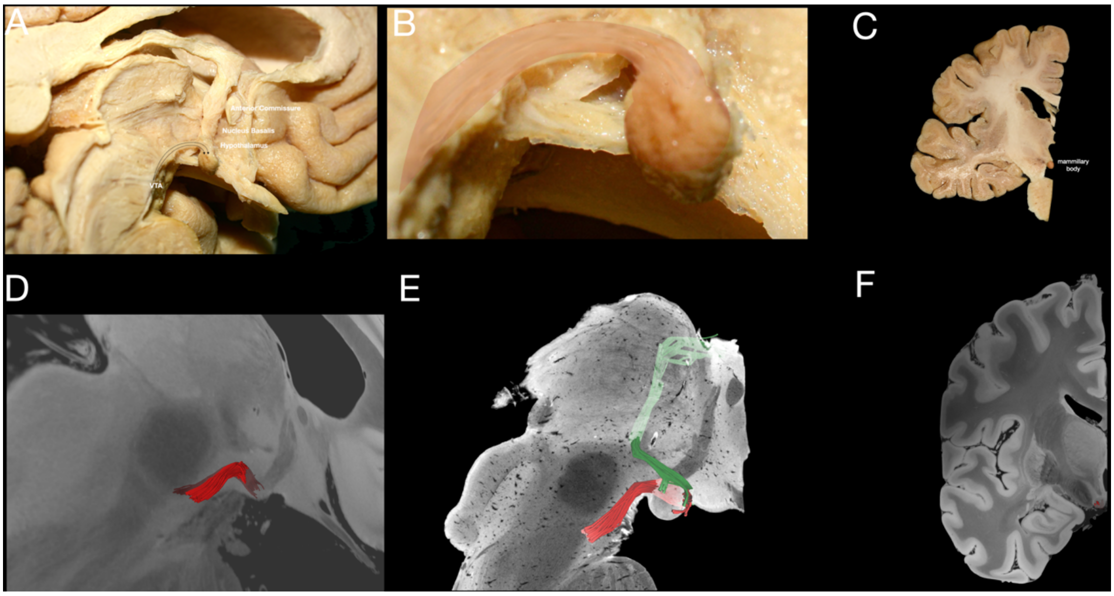

Figure S7. The ventral VMTT. **A.** Medial view of a left hemisphere showing fibers running between the VTA and the mammillary body. **B.** Magnified view of the area demonstrating the trajectory of the fibers highlighted in red. **C.** Coronal section of the contralateral hemisphere at the level of the mammillary body. **D.** Tractography depicting the VTA fibers in red. **E.** Brainstem ex-vivo tractography depicting VTA fibers in red, MTT in green. **F.** Tractography, coronal section revealing VTA fibers within the mammillary body. VMTT, ventral mammillo-tegmental tract.

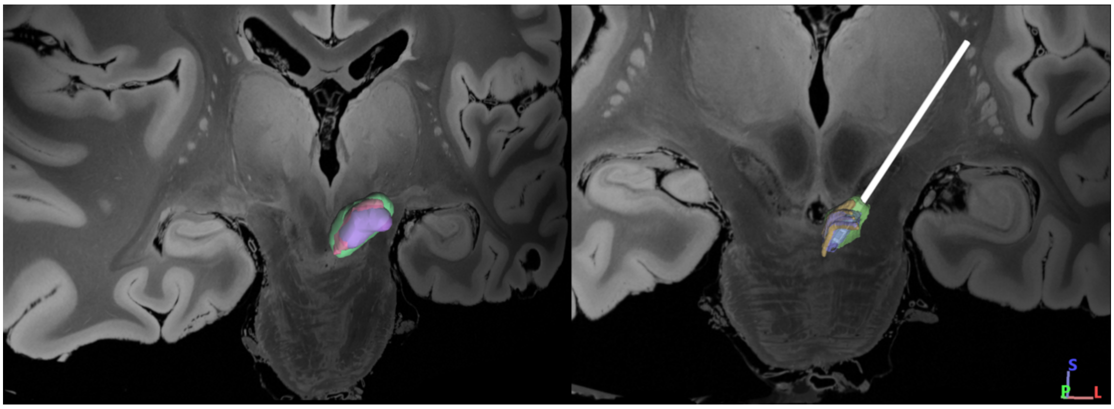

Figure S8 Connectivity-based VTA parcellation. VTA parcellation was achieved according to the regional anatomy of the different VTA tracts of frontal lobe/nucleus accumbens green, insulo-temporal regions orange, and hypothalamus/basal forebrain regions purple.
